# Hypoxic pancreatic stellate cell-derived exosomal mirnas promote proliferation and invasion of pancreatic cancer through the PTEN/AKT pathway

**DOI:** 10.18632/aging.202569

**Published:** 2021-02-26

**Authors:** Wenpeng Cao, Zhirui Zeng, Zhiwei He, Shan Lei

**Affiliations:** 1 Department of Anatomy, School of Basic Medicine, Guizhou Medical University, Guiyang 550009, Guizhou, China; 2 Department of Physiology, School of Basic Medicine, Guizhou Medical University, Guiyang 550009, Guizhou, China; 3 Department of Hepatic-Biliary-Pancreatic Surgery, The Affiliated Hospital of Guizhou Medical University, Guiyang 550009, Guizhou, China

**Keywords:** pancreatic cancer, exosomal microRNAs, PTEN/AKT pathway, metastasis

## Abstract

Pancreatic stellate cells (PSCs) are important components of the tumor microenvironment in pancreatic cancer (PC) and contribute to its development and metastasis through mechanisms that remain incompletely characterized. Tumor hypoxia affects the function and behavior of PC and stromal cells, and can alter exosomal content to modify cell-cell communication. The present study explored the effects of exosomal miRNAs produced by hypoxia-preconditioned PSCs on the growth and metastatic potential of PC cells. Subcutaneous xenografts and liver metastasis mouse models revealed increased tumorigenic potential upon co-implantation of PC cells and PSCs as compared to PC cells alone. Screening miRNA profiles of mouse plasma exosomes and cultured PSCs, followed by miRNA overexpression and inhibition assays, enabled us to identify miR-4465 and miR-616-3p as prominent hypoxia-induced, PSC-derived, exosomal miRNAs promoting PC cell proliferation, migration, and invasion. Proteomics analysis of PC cells incubated with exosomes derived from hypoxic PSCs showed significant downregulation of PTEN. Dual-luciferase reporter assays and western blotting showed that both miR-4465 and miR-616-3p target PTEN and activate AKT signaling in PC cells. We conclude that hypoxia upregulates miR-4465 and miR-616-3p expression in PSC-derived exosomes. Following exosome uptake, these miRNAs promote PC progression and metastasis by suppressing the PTEN/AKT pathway.

## INTRODUCTION

Early metastasis and late tumor detection contribute to making pancreatic cancer (PC) an extremely aggressive malignancy, with a five-year survival rate lower than 5% [1]. Indeed, only 15-20% of patients are diagnosed at an early stage and may benefit from surgical resection, the only potentially curative option for PC. Unfortunately, most patients have recurrence within two years after tumor resection [2, 3]. Therefore, exploring the molecular mechanisms involved in the development of PC is crucial to improve early diagnosis and design more effective therapies.

Pancreatic stellate cells (PSCs) are key stromal components in the tumor microenvironment of PC [4]. PSCs secrete several extracellular matrix components, growth factors, and cytokines and are intimately involved in the metastasis of PC cells. Nan et al. showed that PSCs secrete hepatocyte growth factor (HGF) to activate the c-Met pathway and promote PC cell invasion [5]. In turn, Tang et al. demonstrated that transforming growth factor beta 1 (TGF- β1) enhances PSC-mediated fibrosis in chronic pancreatitis and PC [6]. Several studies revealed that PSC-derived exosomes can deliver proteins, mRNAs, and non-coding RNAs to PC cells to activate oncogenic pathways that enhance proliferation, migration, and drug resistance [7–10]. Various PSC-derived exosomal miRNAs were shown to exert oncogenic roles in PC. Ali et al. in turn identified PSC-derived exosomal miR-221 as a key mediator of PC progression [7]. Very recently, another PSC-derived exosomal miRNA, miR-5703, was shown to promote proliferation of PC cells by downregulating CMTM4 and activating the PI3K/Akt pathway [8]. Takikawa et al revealed that PSC-derived exosomes delivered miR-21-5p and miR-451a to PC cells to promote proliferation, an effect reversed by administration of the exosome inhibitor GW4869 [10].

Due to deficient vascular supply, oxygen levels are markedly decreased in most solid tumors, including PC [11, 12]. Under hypoxic stress, PC and tumor-associated stromal cells change their gene expression profiles to adapt to the microenvironment [13, 14]. However, the mechanisms by which hypoxia modulates communication between stromal and PC cells and ultimately promotes PC progression and metastasis remain inconclusive. In the present study, we examined the changes induced by hypoxia preconditioning of PSCs on exosome-associated miRNA profiles and uncovered a novel oncogenic role for miR-4465 and miR-616-3p on PC, linked to modulation of the PTEN/AKT pathway.

## RESULTS

### PSCs enhance PC growth and metastatic spreading *in vivo*

The effect of PSCs on the proliferation of PC cells *in vivo* was first examined using a subcutaneous tumor model. To this end, PANC-1 cells, alone or combined with human PSCs, were injected subcutaneously into nude mice and tumor volumes recorded over 5 weeks. Results showed that the mice co-injected with PSCs and PANC-1 cells developed larger tumors compared to mice injected with PANC-1 cells alone (Supplementary Figure 1A).

To assess whether PSCs could also enhance the metastatic ability of PC cells, a liver metastasis model was generated by intrasplenic injection of PANC-1 cells, alone or combined with PSCs, into nude mice. Six weeks post-injection, H&E staining revealed significant more metastatic foci in the liver of mice co-injected with the two cell types, compared with those that received PANC-1 cells alone (Supplementary Figure 1B). These data indicate that PSCs can promote both primary tumor growth and metastatic spreading in human PC xenograft models.

### Identification of differentially expressed exosomal miRNAs secreted from hypoxic PSCs

**Figure 1:**
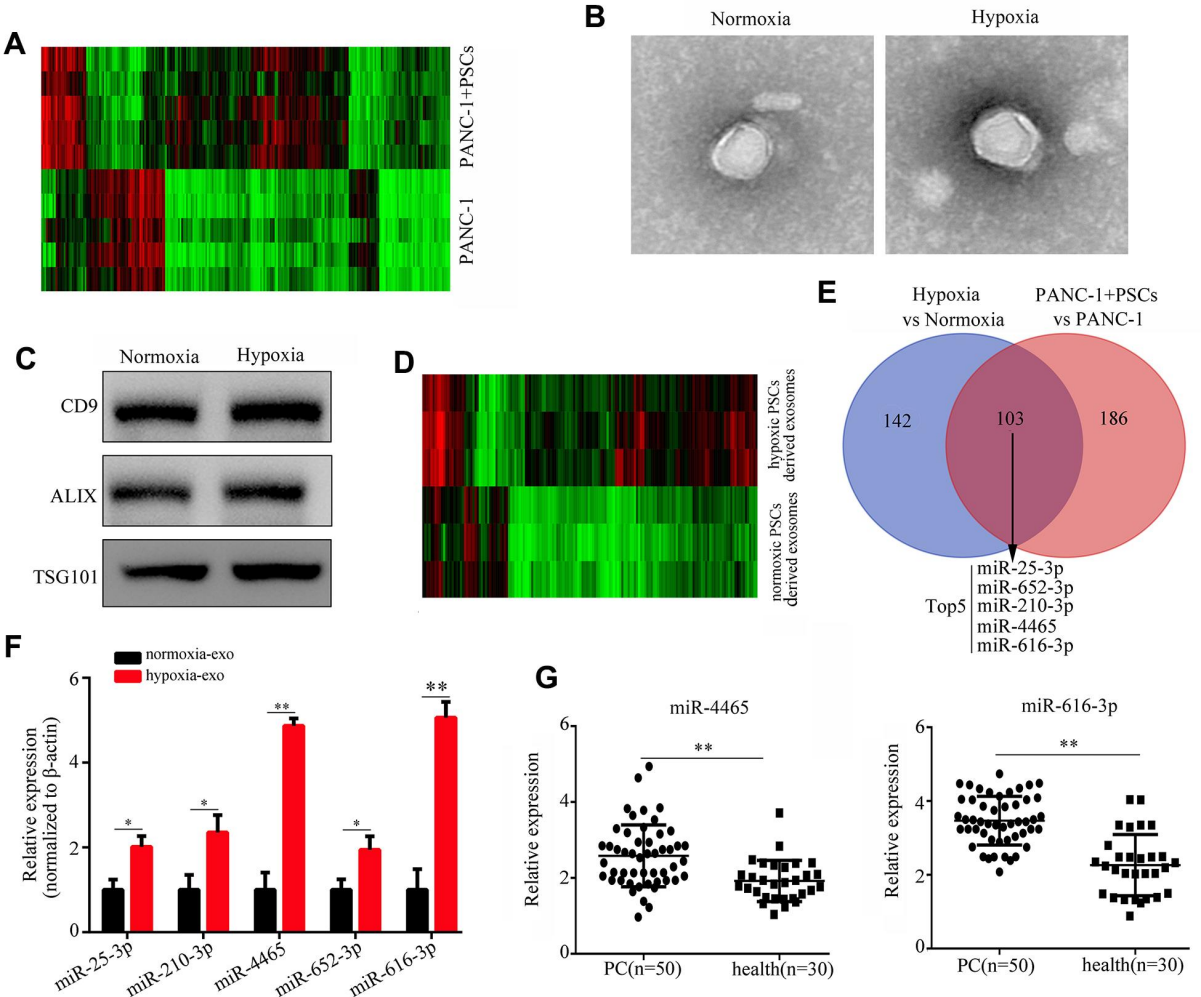
**Hypoxia upregulates PSC-derived exosomal miR-4465 and miR-616-3p.** (**A**) Heatmap displaying differential expression of plasma exosomal miRNAs between mice implanted with PANC-1 cells alone or combined with PSCs. (**B**) TEM images illustrating the morphology of exosomes secreted by hypoxic and normoxic PSCs. (**C**) Western blot detection of exosomal markers in exosomes secreted by hypoxic and normoxic PSCs. (**D**) Heatmap depicting differential expression of exosomal miRNAs in hypoxic vs normoxic PSCs. (**E**) Venn diagram showing the overlap between differentially expressed exosomal miRNAs among *in vivo* and *in vitro* conditions. (**F**) RT-qPCR analysis of miR-25-3p, miR-210-3p, miR-4465, miR-652-3p, and miR-616-3p expression in exosomes produced by hypoxic and normoxic PSCs. (**G**) RT-qPCR analysis of miR-4465 and miR-616-3p expression in plasma exosomes from PC patients (n=50) and healthy controls (n=30). *P<0.05; **P<0.01.

Exosomes were isolated from plasma collected at sacrifice from mice bearing subcutaneous PC xenografts as described above. Exosomal miRNA sequencing revealed 289 miRNAs differently expressed in plasma exosomes from mice co-injected with PANC-1 and PSCs, compared to samples from mice injected with PANC-1 cells alone (Figure 1A). Because the hypoxic microenvironment characteristic of PC can potentially alter the expression profile of exosomal miRNAs derived from tumor-associated cells [15], we contrasted the plasma exosome miRNA profiles recorded above with those from PSCs cultured under hypoxia and normoxia. The exosomes collected from cultured PSCs consisted of lipid-bilayer membranous vesicles on TEM and expressed exosomal markers, including CD9, ALIX, and TSG101 (Figure 1B, 1C). Results of miRNA sequencing showed that 245 miRNAs were differently expressed in exosomes secreted from PSCs grown under hypoxia, compared to normoxia-incubated cells (Figure 1D).

Next, the corresponding *in vivo* (plasma-derived) and *in vitro* miRNA profiles were compared to identify common transcripts differentially expressed among conditions. A total 103 specific hypoxic PSCs-derived exosomal miRNAs were shown to overlap with those registered in plasma exosomes. Among these, miR-25-3p, miR-625-3p, miR-210-3p, miR-4465, and miR-616-3p showed the greatest differential expression (Figure 1E). We further used qRT-PCR to detect the expression of these miRNAs in exosomes from PSCs cultured under normoxia and hypoxia. Results showed that the expression of both miR-4465 and miR-616-3p increased most significantly (>3 fold) after hypoxic exposure (Figure 1F). Moreover, we found that the expression of miR-4465 and miR-616-3p was significantly increased in plasma exosomes from PC patients (n=50), compared with healthy individuals (n=30) (Figure 1G).

### Hypoxic PSC exosome-derived miR-4465 and miR-616-3p promote proliferation and migration of PC cells

**Figure 2:**
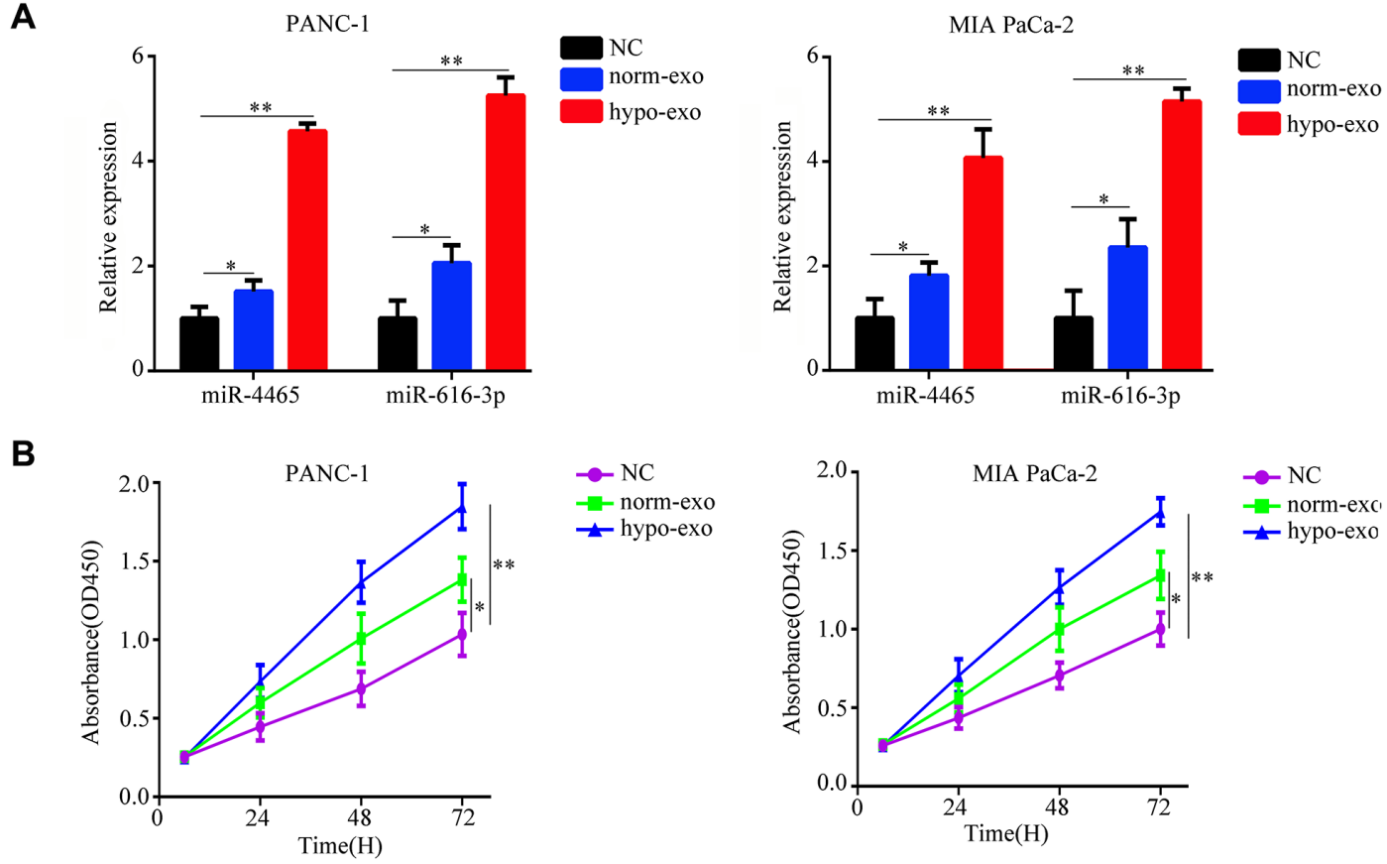
**Hypoxic PSC-derived exosomal miR-4465 and miR-616-3p act on PC cells to stimulate proliferation.** PANC-1 and MIA PaCa-2 cells were treated with exosomes secreted from hypoxic or normoxic PSCs. EV-depleted complete medium was used as control. (**A**) RT-qPCR analysis of miR-4465 and miR-616-3p expression in PC cells. (**B**) CCK-8 proliferation assay results. *P<0.05; **P<0.01.

**Figure 3:**
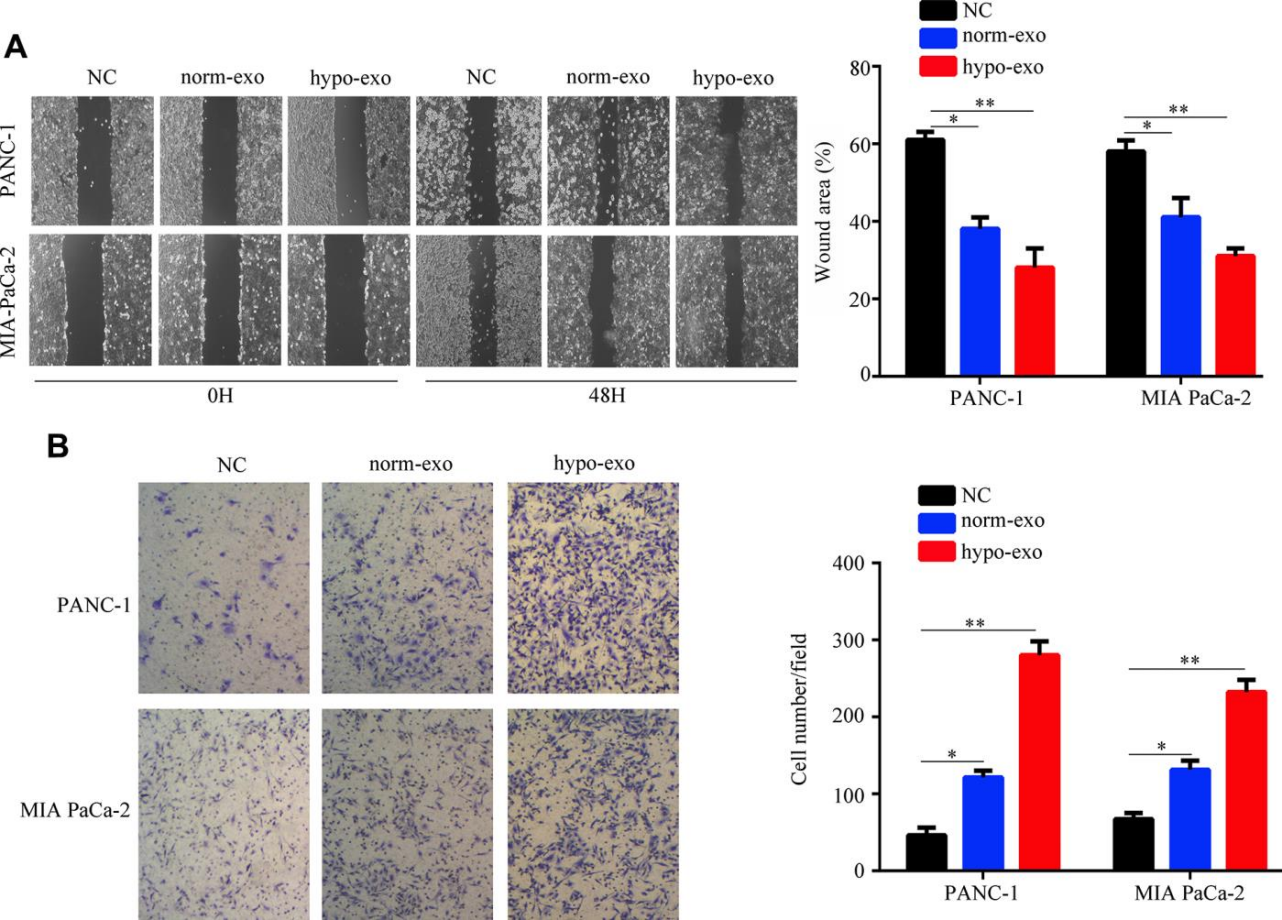
**Hypoxic PSC-derived exosomal miR-4465 and miR-616-3p promote migration and invasion in PC cells.** PANC-1 and MIA PaCa-2 cells were treated with exosomes secreted from hypoxic and normoxic PSCs. EV-depleted complete medium was used as control. (**A**) Results of wound-healing migration assays (magnification: 40×). (**B**) Results of Transwell invasion assays (magnification: 40×). *P<0.05; **P<0.01.

We next tested whether PSC-derived exosomes can deliver miR-4465 and miR-616-3p to PC cells. Results showed that exposure to both normoxic and hypoxic PSC-derived exosomes increased the expression of miR-4465 and miR-616-3p in both PANC-1 and MIA PaCa-2 PC cells; however, for both miRNAs the upregulation was stronger in cells incubated with exosomes derived from hypoxic PSCs (Figure 2A). Next, the CCK-8 assay was used to detect the effect of PSC-derived exosomes on the proliferation of PC cells. Results showed that hypoxic PSC-derived exosomes significantly increased the proliferation of both PANC-1 and MIA PaCa-2 cells, compared with cultures treated with either normoxic PSC-derived exosomes or exosome-depleted medium, used as negative control (Figure 2B). Furthermore, both wound healing and Transwell assays revealed that treatment with hypoxic PSC-derived exosomes significantly enhanced migration and invasion in both cell lines (Figure 3A, 3B).

**Figure 4:**
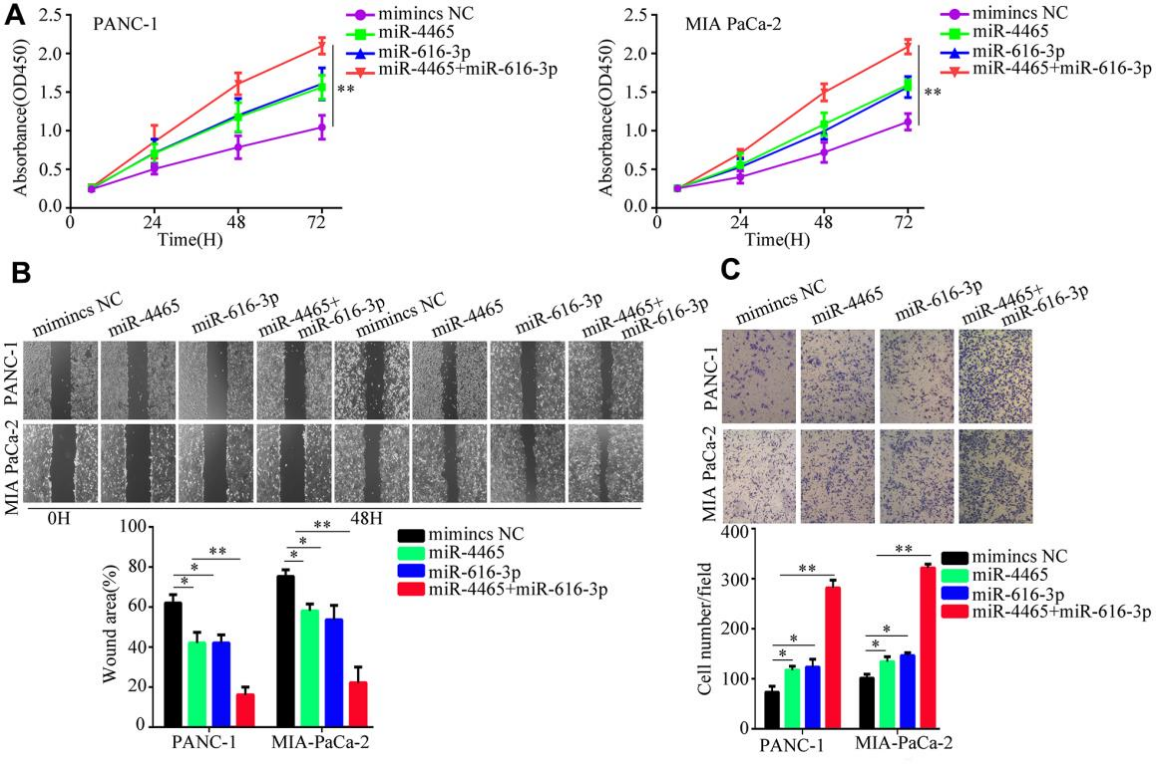
**miR-4465 and miR-616-3p overexpression enhances PC cell proliferation, migration, and invasion.** PANC-1 and MIA PaCa-2 were transfected with miR-4465, miR-616-3p, or negative control (NC) mimics. (**A**) CCK-8 proliferation assay results. (**B**) Wound-healing migration assay results. (**C**) Transwell invasion assay results. *P<0.05; **P<0.01.

**Figure 5:**
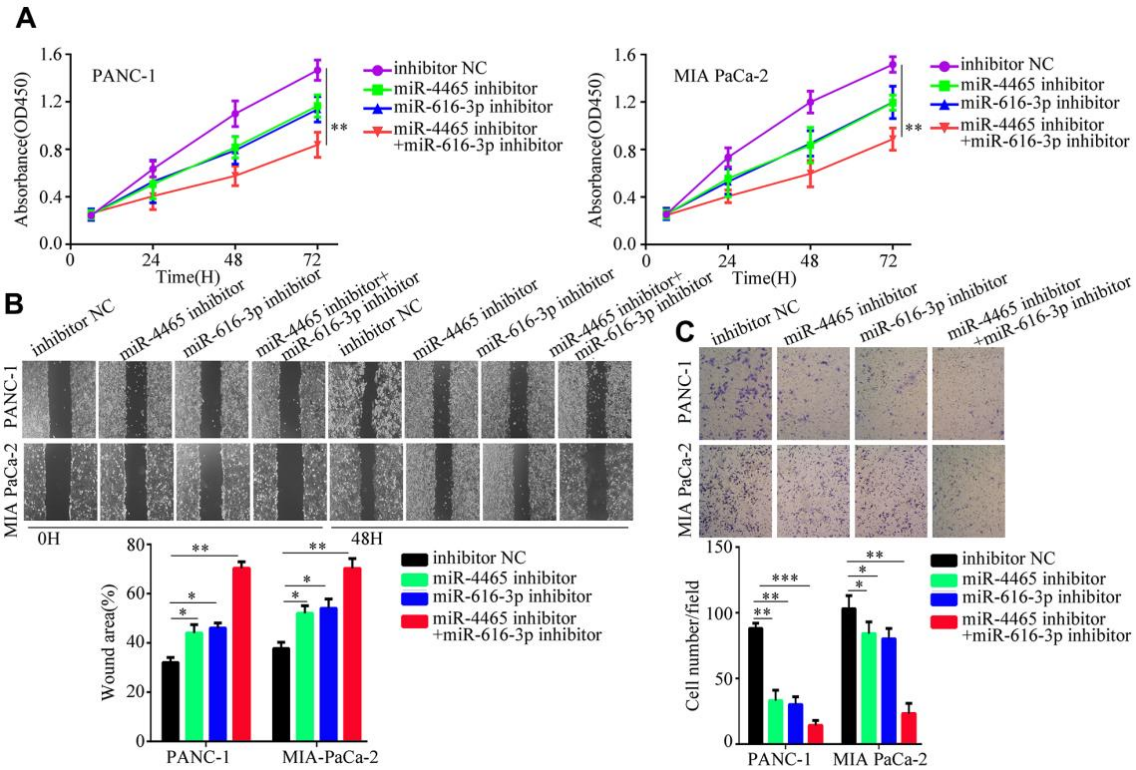
**MiR-4465/miR-616-3p inhibition abrogates PSC exosome-induced proliferation, migration, and invasion of PC cells.** PANC-1 and MIA PaCa-2 cells were transfected with inhibitors of miR-4465 and/or miR-616-3p, or with the corresponding negative controls (NC), prior to treatment with hypoxic PSC-derived exosomes. (**A**) Results of CCK-8 proliferation assays. (**B**) Results of wound-healing migration assays. (**C**) Results of Transwell invasion assays. *P<0.05; **P<0.01.

To confirm that the proliferation and migration/invasion potential of PC cells was indeed stimulated by an increase in intracellular miR-4465 and miR-616-3p levels, we evaluated these processes in PANC-1 and MIA PaCa-2 cells transfected with miR-4465 and/or miR-616-3p mimics. Results showed that each single miRNA mimic enhanced proliferation, migration, and invasion in both cell lines, while co-transfection with the two miRNA mimics further enhanced these effects (Figure 4A–4C). To further validate these findings, we transfected PC cells with miR-4465 and/or miR-616-3p inhibitors before treatment with hypoxic PSC-derived exosomes. While both inhibitors independently decreased the effect of hypoxic PSC-derived exosomes on PC cell proliferation, combined transfection resulted in a stronger effect (Figure 5A). In turn, pre-treatment of PC cells with miR-4465 and miR-616-3p inhibitors reversed the stimulation of migration and invasion exerted by hypoxic PSCs-derived exosomes, whereas a stronger repression was noted upon combining both inhibitors (Figure 5B, 5C).

### PTEN is a target of miR-4465 and miR-616-3p

**Figure 6:**
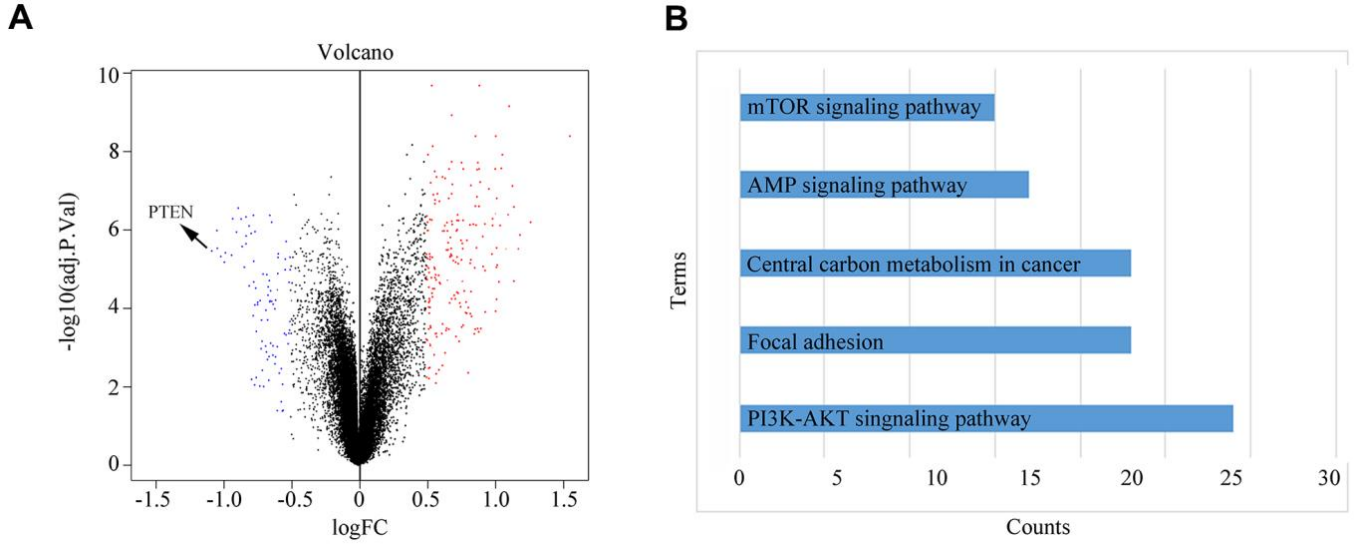
**Differential protein expression and KEGG pathway analysis.** (**A**) PANC-1 cells were treated with normoxic or hypoxic PSC-derived exosomes prior to iTRAQ-based analysis of protein profiles. (**B**) KEGG analysis results showing the top-5 pathways enriched in the differentially expressed proteins identified in PANC-1 cells treated with hypoxic PSC-derived exosomes.

To explore the molecular mechanism by which hypoxic PSC-derived exosomal miR-4465 and miR-616-3p stimulate growth and migration in PC cells, we treated PANC-1 cells with normoxic and hypoxic PSC-derived exosomes and performed iTRAQ analysis to determine protein profiles. Results showed that upon treatment with hypoxic PSC-derived exosomes PTEN was the most significantly downregulated protein (Figure 6A). KEGG analysis showed that the differentially expressed proteins identified by iTRAQ analysis were significantly enriched in five signaling pathways, namely PI3K-AKT, mTOR, central carbon metabolism, cAMP, and focal adhesion (Figure 6B).

**Figure 7:**
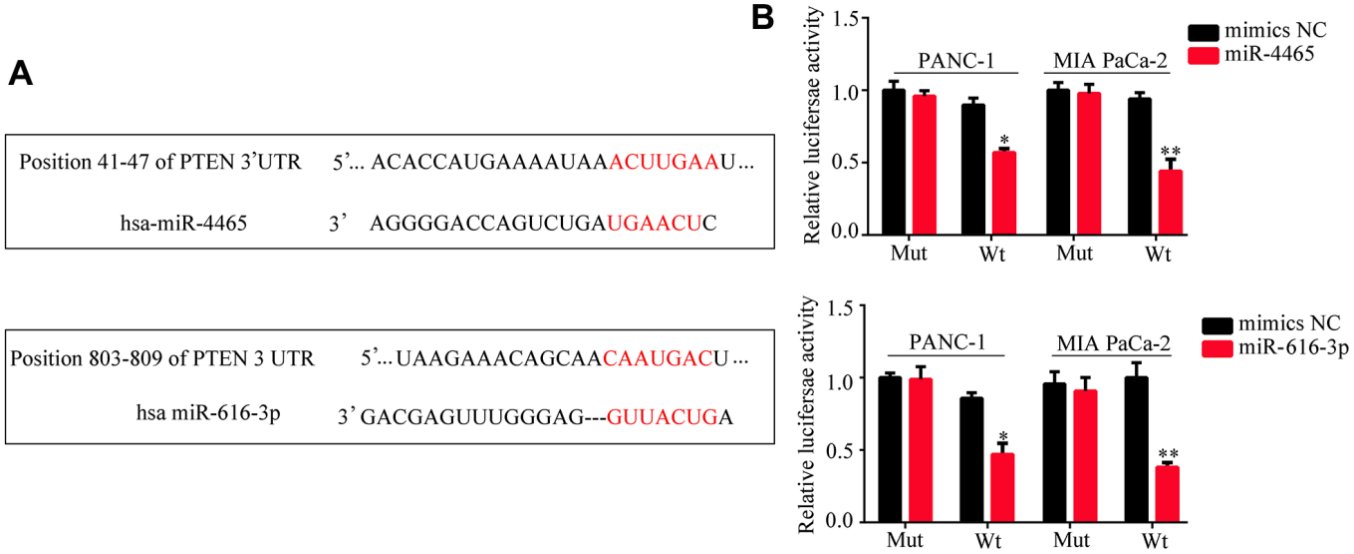
**PTEN is a target of miR-4465 and miR-616-3p.** (**A**) Identification of PTEN as a miR-4465 and miR-616-3p target by TargetScan. (**B**) Dual-luciferase reporter assay results indicating interaction between both miR-4465 and miR-616-3p and the WT 3’UTR of PTEN.

**Figure 8:**
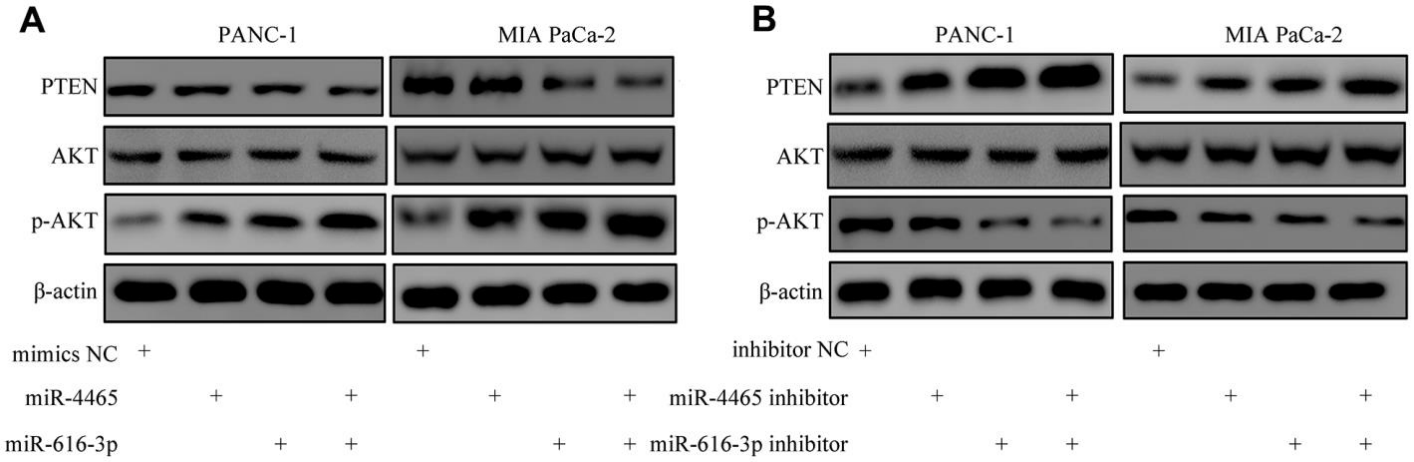
**MiR-4465 and miR-616-3p downregulate PTEN and activate AKT in PC cells.** (**A**) Western blot analysis of PTEN, AKT, and p-AKT expression in PANC-1 and MIA PaCa-2 cells transfected with miR-4465, miR-616-3p, or negative control (NC) mimics. (**B**) Western blot analysis of PTEN, AKT, and p-AKT expression in PANC-1 and MIA PaCa-2 cells transfected with miR-4465 inhibitor, miR-616-3p inhibitor, or the corresponding negative controls (NC) prior to treatment with hypoxic PSC-derived exosomes. *P<0.05; **P<0.01.

We then accessed TargetScan to identify putative mRNA targets of miR-4465 and miR-616-3p. Results showed that both miRNAs can bind PTEN mRNA (Figure 7A). To verify these interactions, we conducted dual-luciferase reporter assays. We found that overexpression of either miR-4465 or miR-616-3p significantly reduced the luciferase activity associated with the WT 3′-UTR of the PTEN transcript, whereas no significant reduction was observed in cells transfected with a mutant (MUT) 3′-UTR sequence (Figure 7B). Meanwhile, western blotting showed that transfection with miR-4465 or miR-616-3p mimics decreased the expression of PTEN and increased the expression of p-AKT, while the combination of both miRNA mimics potentiated these effects (Figure 8A). Subsequently, we used inhibitors of miR-4465 and miR-616-3p to treat PC cells prior to exposure to hypoxic PSC-derived exosomes. Western blot results demonstrated that both inhibitors were independently able to increase PTEN and reduce p-AKT expression, while their combination accentuated these effects (Figure 8B). Taken together, these results demonstrated that hypoxic PSC-derived exosomal miR-4465 and miR-616-3p target PTEN and activate the PTEN/AKT pathway.

## DISCUSSION

Despite therapeutic advances, early metastasis and late tumor detection contribute to the extremely poor prognosis of patients with PC [16]. Hypoxia is a critical factor promoting communication between PC and stromal cells in the tumor microenvironment [17, 18]. On the other hand, mounting evidence suggests that exosomal miRNAs derived from PSCs are key mediators of PC progression and metastatic dissemination [8, 10]. Therefore, the present study examined the effects of hypoxia on the expression of miRNAs carried by PSC-derived exosomes and analyzed their impact on PC cell growth, migration, and invasion. We found that miR-4465 and miR-616-3p were the most upregulated miRNA transcripts within exosomes isolated from hypoxia-preconditioned PSCs. Importantly, we discovered also that both miRNAs were significantly overexpressed in plasma exosomes from patients with PC, compared with those of healthy individuals.

MiRNAs have been identified as vital regulators of biological functions and can play oncogenic or tumor suppressive roles in different tumors [19]. MiR-4465 expression has been positively associated with tumor proliferation and chemotherapy resistance in breast cancer, and shown to be negatively regulated by neoadjuvant bevacizumab therapy [20]. However, in non-small cell lung cancer miR-4465 was suggested to play a suppressive role by decreasing the expression of the oncogene EZH2 [21]. MiR-616-3p was shown to promote proliferation and metastasis in gastric cancer via targeting PTEN [22]. In the current study, we showed that exosomes released by hypoxic PSCs delivered miR-4465 and miR-616-3p to PC cells to promote proliferation and metastatic capacity. These effects were reproduced by overexpression of miR-4465 and miR-616-3p in PC cells and reversed by pre-treatment with the corresponding inhibitors. Thus, our study provides for the first time evidence for an oncogenic role of hypoxia-related, PSC-derived exosomal miR-4465 and miR-616-3p in PC.

KEGG analysis showed that the differentially expressed proteins identified by iTRAQ in PC cells treated with exosomes from hypoxia-preconditioned PSCs were significantly enriched in the PI3K-AKT signaling pathway. This pathway stimulates cancer progression by regulating a series of biological processes, including proliferation, invasion, and drug resistance [23, 24]. A highly downregulated protein in PC cells exposed to hypoxic PSC-derived exosomes was PTEN, which functions as a tumor suppressor in various cancers, including PC, by antagonizing the activation of the PI3K/AKT pathway [25, 26]. Importantly, we showed that PTEN is a target of both miR-4465 and miR-616-3p and that overexpression of either miRNA in PC cells decreased PTEN expression and activated AKT. Accordingly, incubation of PC cells with miR-4465 and/or miR-616-3p inhibitors prior to treatment with hypoxic PSC-derived exosomes rescued PTEN expression and inhibited AKT activation.

In summary, the present study demonstrated that hypoxia upregulates the expression of miR-4465 and miR-616-3p in exosomes released by PSCs *in vitro* and possibly *in vivo*. These exosomal miRNAs can be transferred to PC cells to promote proliferation and invasion via PTEN repression and subsequent AKT activation. Importantly, we showed that miR-4465 and miR-616-3p are also upregulated in plasma exosomes from PC patients. Along with the present experimental findings, this evidence suggests that these miRNAs might serve as diagnostic biomarkers and/or therapeutic targets. Our study has, however, some limitations. Even though we provided robust evidence for a pro-oncogenic role of hypoxia-induced, PSC-derived exosomal miRNAs in PC, their potential impact on PTEN expression and PC progression and metastasis *in vivo* is still uncertain. These caveats could be adequately addressed through inhibition and overexpression of candidate miRNAs in PSCs used to generate animal PC models.

## MATERIALS AND METHODS

### Blood sampling procedure

Patients diagnosed with PC between March 2018 and January 2020 at the Affiliated Hospital of Guizhou Medical University (n = 50) and healthy individuals (n = 30) participated in the present study. All patients provided 20 mL of blood, collected in EDTA-anticoagulant tubes. Blood samples were centrifuged within 1 h from collection to separate plasma and blood cells. Plasma was stored at -80° C for downstream analyses. The current study was performed in accordance with the Declaration of Helsinki and was approved by the Ethical Committee of Guizhou Medical University. All patients enrolled in the present study signed written informed consent forms.

### Cell culture and transfection

Human PC cell lines PANC-1 and MIA PaCa-2 were purchased from ATCC (USA). Human pancreatic stellate cells (PSCs) were obtained from National Infrastructure of Cell Line Resource (http://www.cellresource.cn/). All cells were cultured in DMEM with 10% extracellular vesicle (EV)-depleted FBS at 37° C in a humidified atmosphere with 5% CO_2_. To simulate hypoxia, PSC cultures were maintained for 24 hs in a nitrogen-filled incubator (94% N_2_, 5% CO_2_, 1% O_2_). MiR-4465 mimics, miR-616-3p mimics, miR-4465 inhibitor, miR-616-3p inhibitor, and corresponding control mimics and inhibitors were obtained from Sangon Biotech (Shanghai, China). The transfection processes were conducted using Lipo2000 (RIBOBIO, Guangzhou, China) according to the manufacturer's instructions.

### Animal experiments

Ten female BALB/c nude mice (6 weeks old) were employed to generate the subcutaneous tumor model. A total of 1 ×10^7^ PANC-1 cells, alone or admixed with 1 ×10^5^ human PSCs, were injected subcutaneously into the left armpit of mice (n = 5 mice per group). After 5 weeks, the mice were sacrificed and tumors were dissected and weighed. Blood samples were also collected and separated to obtain plasma. For metastasis analysis, a liver metastasis model was generated by injecting 100 μL PBS containing 1×10^7^ PANC-1 cells, alone or admixed with 1×10^5^ human PSCs, into the spleen of nude mice (n = 5 mice per group). After 6 weeks, the mice were sacrificed and liver tissues dissected and stained with H&E to detect metastatic foci. All animal experiments were approved by the Animal Ethics Committee of Guizhou Medical University.

### Isolation of exosomes and exosomal RNAs

Exosomes from human and mouse plasma were extracted using an Invitrogen™ Total Exosome Isolation Kit (ThermoFisher, USA) [27]. Briefly, 200 uL of plasma was mixed with the precipitation reagent provided by the kit and exosomes were isolated according to the manufacturer’s instructions. The exoRNeasy Serum/Plasma Maxi Kit (Yeasen Biotech, Shanghai, China) [28] was employed to isolate exosomal RNAs in samples previously spiked with 25 fmol of C. elegans cel-miR-39 standard RNA (Sangon, Shanghai, China), used as control. The exosomes were stored at -80° C before cell culture experiments and molecular analyses.

### Transmission electron microscopy (TEM)

The morphology of exosomes isolated from plasma was examined using TEM. After fixation in 1% glutaraldehyde at room temperature and washing with deionized water, the exosomes were placed on formvar/carbon-coated 300 mesh copper grids (Servicebio, Wuhan, China). Exosomes were then stained with 2% uranyl oxalate, washed in PBS, air dried at room temperature, and photographed using a JIB-4700F TEM (JEOL Japan Electronics Co., Ltd., Japan).

### miRNA sequencing

Exosomal miRNAs were sequenced on an Illumina HiSeq 2000 platform (Illumina, USA). Low expression miRNAs with mean fragments per kilobase million<1 were removed before analyze. After normalization, miRNA expression profiles were analyzed using ANOVA. Log2 fold change >1 and adjusted P-value 0.05 were set as cut-off for statistical significance. Differential miRNA expression was visualized as a heatmap.

### Western blotting

Total proteins were extracted from PC cells using RIPA reagent containing 5% PMSF protease inhibitor (Boster, Wuhan, China). The BCA method was used to estimate protein concentrations. Proteins (30 μg per sample) were separated by 10% SDS-PAGE for 120 min and then transferred onto 0.45 μm pore size PVDF membranes (Millipore, USA). The membranes were then blocked using 5% non-fat milk and incubated for 12 h at 4° C with primary antibodies against PTEN, AKT, p-AKT, and β-actin (all from Proteintech, Wuhan, China). High sensitivity ECL reagent was used to visualize the blots in a Multi Imager (Bio-Rad, USA). Relative protein expression was calculated using ImageJ (NIH, USA). β-actin and AKT were used as references for PTEN and p-AKT, respectively. Antibodies against CD9, ALIX, and TSG101 (all from Proteintech, Wuhan, China) were used to positively identify exosomes.

### Real-time fluorescence-based quantitative PCR

Exosomal RNAs or total miRNAs in cells were reversely transcribed using a miRNA First Strand cDNA Synthesis (Tailing Reaction) Kit and quantified using SYBR Green reagent (both from Sangon Biotech, Shanghai, China). The cel-miR-39 [29, 30] was used as internal reference for exosomal miR-4465 and miR-616-3p, and U6 snRNA was used as internal reference for cellular miR-4465 and miR-616-3p. The sequence of the miR-4465 primer was 5'-CUCAAGUAGUCUGAC-3'. The sequence of the miR-616-3p primer was 5'- ACACTCCAGCTGGGAGTCATTGGAGGGTTT-3'. The sequence of the U6 primer was 5'-CGCTTCGGCAGCACATATAC-3'. The sequence of cel-miR-39 primer was 5'-UCACCGGGUGUAAAUCAGCUUG-3'.

### Cell proliferation assay

PANC-1 and MIA PaCa-2 cells were seeded into 96-well plates at a density of 5×10^3^ cells/well (6 replicates per treatment). After 24, 48, and 72 h, 100 μl of DMEM containing 10 μl CCK-8 reagent (Boster Bio, Wuhan, China) was added to each well. The optical density (OD) of each well was measured at 450 nm.

### Wound healing assay

A total of 5×10^5^ PANC-1 or MIA PaCa-2 cells per well were seeded into 6-well plates. When confluence reached 100%, a sterile 200 μl micropipette tip was used to scratch the cell monolayers. Floating cells were washed out with PBS and adherent cells were cultured for 48 h in DMEM without FBS. Images were taken at 0 and 48 h on an optical microscope (Olympus, Japan) and migrating distance calculated using ImageJ.

### Cell invasion assay

Transwell chambers (8-μm pore size; Corning, USA) pre-coated with Matrigel (Corning, USA) were used to evaluate the invasive ability of PC cells. Serum-free DMEM (300 μl) containing 2×10^4^ PANC-1 or MIA PaCa-2 cells was added into the upper chamber. DMEM containing 10% FBS (700 μl) was added to the lower chamber as chemoattractant. After culturing for 24 h at 37° C, the cells were fixed in 4% paraformaldehyde and stained with 1% crystal violet. After removing non-invasive cells, images from five random fields of view were captured in an optical microscope (Olympus, Japan) and invasive cells were counted using ImageJ.

### iTRAQ protein profile analysis

Following exposure to hypoxic/normoxic PSC-derived exosomes or control medium, PC cells were lysed using 8 M carbamide, 30 mM HEPES, 1 mM PMSF, 2 mM EDTA, and 10 mM DTT. After centrifuging at 8,000 g at 4° C for 15 min, total protein was obtained and digested into peptides. The peptides were resuspended in 0.5 M triethylammonium bicarbonate and labeled using iTRAQ (isobaric tags for relative and absolute quantitation) reagent. After vacuum drying, iTRAQ-labeled peptides were desalted using Strata X C18 SPE columns (Phenomenex, USA) and analyzed on a Q-Exactive mass spectrometer (ThermoFisher Scientific, USA). Log fold-change >0.5 and P-value <0.05 were set as thresholds for differential protein expression.

### Dual-luciferase reporter assay

PTEN was predicted as a target of miR-4465 and miR-616-3p by TargetScan. PTEN plasmids containing wild type (Wt) or mutant (Mut) binding sites for miR-4465 or miR-616-3p were constructed and sub-cloned into the psiCHECK-2 vector (Promega, USA), which contains a constitutively expressed firefly luciferase gene. Control and miR-4465- or miR-616-3p- overexpressing PANC-1 and MIA PaCa-2 cells were then transfected with the above PTEN luciferase reporter vectors. Luciferase activity was measured using a Dual-Luciferase Reporter Assay System (Promega, USA).

### Data analysis

All data were analyzed using SPSS 20.0 software (IBM Corp., USA). ANOVA with Bonferroni’s post hoc test was used to analyze differences between multiple groups. P<0.05 was set as threshold for statistical significance.

### Data availability

Data supporting the findings of this study are available from the corresponding author upon request.

## Supplementary Material

Supplementary Figure 1
