# Supplementary material for: Hypoxic pancreatic stellate cell-derived exosomal mirnas promote proliferation and invasion of pancreatic cancer through the PTEN/AKT pathway
Source: Aging (Albany NY). 2021 Feb 26;13(5):7120–32. doi: 10.18632/aging.202569 (PMC7993707; doi:10.18632/aging.202569)
Supplement: Supplementary Figure 1 [file aging-13-202569-s001.pdf]

SUPPLEMENTARY FIGURE

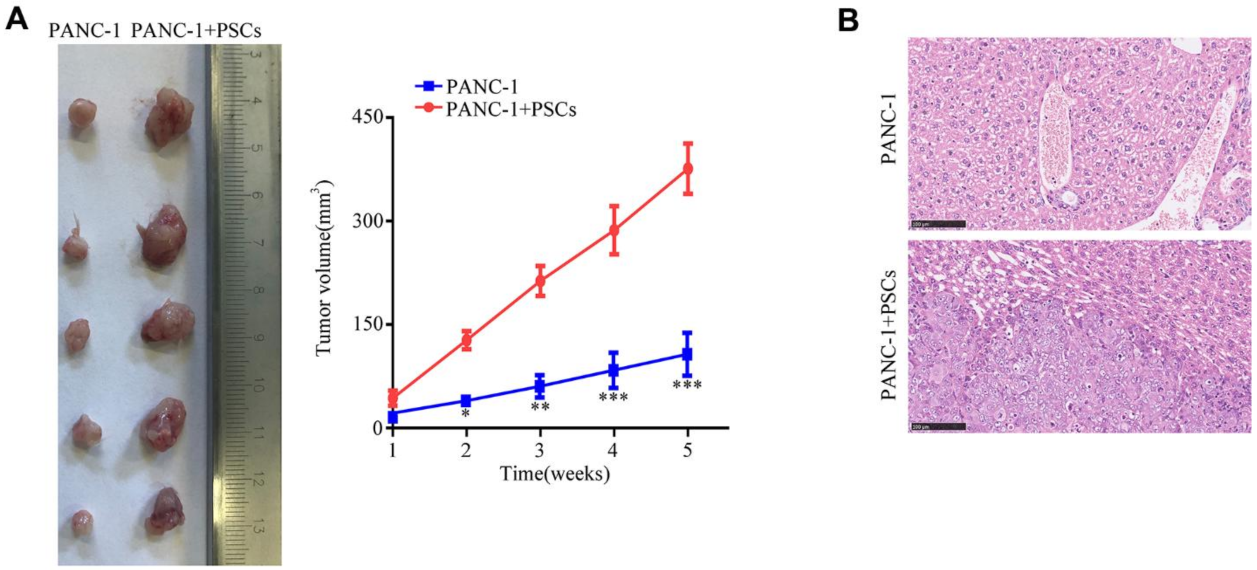

Supplementary Figure 1. PSCs promote proliferation and metastasis of PC cells *in vivo*. (**A**) Tumor volume measurements. Subcutaneous xenografts were produced in nude mice by injecting PANC-1 cells alone or in combination with PSCs. (**B**) Liver H&E staining images showing a higher number of metastatic foci following intrasplenic co-injection of PANC-1 and PSCs. *P<0.05; **P<0.01.
